# Food Simulating Organic Solvents for Evaluating Crosslink Density of Bulk Fill Composite Resin

**DOI:** 10.1155/2017/1797091

**Published:** 2017-04-12

**Authors:** Neveen M. Ayad, Hala A. Bahgat, Eman Hussain Al Kaba, Maryam Hussain Buholayka

**Affiliations:** ^1^Restorative Dental Sciences Department, College of Dentistry, University of Dammam, Dammam, Saudi Arabia; ^2^College of Dentistry, University of Dammam, Dammam, Saudi Arabia

## Abstract

*Objectives.* To evaluate crosslink densities of two bulk fill composite resins and determine if the used Food Simulating Organic Solvent (FSOS) affected them.* Methods.* Forty specimens were prepared from SureFill and SonicFill bulk fill composite resins, 20 each. All specimens were stored dry for 24 h. Each group was divided into 2 subgroups: stored in ethanol (E) 75% or in methyl ethyl ketone (MEK) 100% for 24 h. Crosslink density was evaluated by calculating the difference between the Vickers hardness numbers of the specimens stored dry and after their storage in FSOS. The data were statistically analyzed using *t*-test.* Results.* The means of crosslink density in E and MEK were 6.99% and 9.44% for SureFill and 10.54% and 11.92% for SonicFill, respectively. *t*-test displayed significant differences between crosslink densities of SureFill and SonicFill: (*P* < 0.0001) in E and (*P* = 0.02) in MEK and between crosslink densities of SureFill in E and MEK (*P* = 0.02).* Conclusions.* Crosslink density of bulk fill composite resin can be evaluated using E or MEK. SureFill has higher crosslink density than SonicFill in both E and MEK.

## 1. Introduction

The oral cavity contains a diversity of chemical components from food and saliva including alkalis, acids, salts, and alcohol that may be absorbed by composite resin resulting in their degradation [1–3]. Water which is a major constituent of human saliva has a proven influence on composite resin [4–6]. Ethanol (E) is an organic solvent that is considered a food simulant by the US Food and Drug Administration (FDA) [7]. Methyl ethyl ketone (MEK) which naturally exists in various types of fruits, meat, vegetables, and yogurt has also been approved by the FDA as a food additive and is considered a food simulating liquid [8].

To evaluate the softening effect of the oral environment on composite resin, the solubility parameter is of extreme importance. It provides a numerical value through which the level of interaction between materials can be expected [4–9]. Greater softening effects are expected when the resin matrix and the solvent have closely matching solubility parameters [10–12].

Both ethanol and methyl ethyl ketone FSOS have solubility parameters closely matching that of Bis-GMA (the most commonly used monomer in composite resin) [9]. Previous studies relied on ethanol, either in its absolute form or diluted 75% E/water [13], to evaluate the crosslink density of the polymer network.

Bulk fill composite resin is widely spreading amongst dentists due to its simple application technique. It displayed adequate light-curing to about 5 mm depth by measuring degree of conversion, compressive strength, and top/bottom hardness [14]. However, its durability during service can be affected not only by how efficient its curing was which directly affects the extent of monomer transformation into polymer, but also to which extent the polymer network is crosslinked. The degree of conversion is the number of ethylene double carbon bonds that are converted into single bonds of the composite resin to obtain the optimal chemical-physicomechanical behavior. However, polymers with comparable degree of conversion may have dissimilar crosslink densities due to variances in the linearity/branching of the polymer chains [15].

Recent studies have proved that MEK has stronger softening effect on bulk fill composite resin than E [2]. Thus the authors hypothesize that MEK could be an efficient alternative for E in assessment of crosslink density of bulk fill composite resin.

## 2. Materials and Methods

Two bulk fill composite resins were used in this investigation (Table 1).

### 2.1. Specimens' Preparation

Twenty specimens were prepared from each composite resin; 10 were kept in E and the other 10 in MEK. The bulk fill composite resin was packed into a cylindrical Teflon mold of 2.5 mm inner diameter and 5 mm height placed on a glass slab. The mold was packed with composite resin as a single layer. SonicFill was applied by sonic activated hand-piece, as recommended by the manufacturer. Light-irradiation was performed for 20 s on the top of the specimens using a light emitting diode curing unit (1200 mW/cm^2^, Bluephase 20i, Ivoclar Vivadent, Schaan, Liechtenstein). The top surfaces of all specimens were marked. Then the specimens were kept dry for 24 h at room temperature (23 ± 1°C) before testing.

### 2.2. Evaluation of Crosslink Density

The evaluation of crosslink density was determined indirectly by calculating the difference between the top Vickers hardness numbers of the specimens stored dry for 24 h and after their storage in FSOS [15].

#### 2.2.1. Measurement of Vickers Hardness Number of the Specimens Stored Dry for 24 h

Three indentations (load: 50 g; dwell time: 15 seconds) were performed on the top surface of each specimen using Vickers's hardness tester (Micromet 6049, Buehler, Illinois, USA). The mean of these three individual measurements was considered as the Vickers hardness number of the overall surface.

#### 2.2.2. Measurement of Vickers Hardness Number after Specimens' Storage in FSOS

The specimens were stored individually in 10 mL of either 75% ethanol or 100% methyl ethyl ketone at 37 ± 1°C according to their groups for 24 h. A remeasurement of the Vickers hardness number of the top surface was recorded for the specimens removed from the respective FSOS. The crosslink density was obtained by using the formula [15]:

(1) Crosslink density=Vickers hardness number after storage in FSOS−Vickers hardness number after 24 h of dry storage100.

### 2.3. Statistical Analysis

Statistical data were analyzed by using SPSS-20.0 (IBM product-USA). Mean and standard deviation were calculated for the Vickers hardness number of all subgroups. Two way ANOVA was used to assess the overall effect of material and solvent on the crosslink density and the estimated means were compared using Bonferroni adjustment. *t*-test was used to assess differences in crosslink density after using different solvents in each material and between the two materials after the use of each solvent.

## 3. Results

The mean Vickers hardness numbers of the tested bulk fill composite resins stored dry for 24 h and stored in E and MEK as well as the calculated crosslink densities are presented in Table 2. Two way ANOVA displayed a significant effect of the bulk fill composite resin and the Food Simulating Organic Solvent on the crosslink density. The estimated means were compared using Bonferroni adjustment. *t*-test displayed significant difference between the crosslink density of SureFill and SonicFill for each used FSOS. *t*-test also revealed significant difference between the crosslink densities measured for SureFill specimens stored in E and MEK.

## 4. Discussion

Crosslink density is an important indicator of the chemical stability of composite resin [12, 16]. A polymer with high crosslink density is characterized by tight network structure with improved packing density and minimized free vicinity of the entangled polymer chains. This restricts the diffusion of the solvent molecules in and out of the polymer structure and as a result decreases their plasticization impact [12]. Consequently, the magnitude of crosslink points in a resin structure dictates its mechanical performance. A limited crosslinked resin structure shows less stiffness, more rubbery behavior, and poor surface integrity [17]. Surface hardness is considered a key performance indicator of the service life of composite resin dental restorations [1]. Softening tests, that are based on evaluating the top hardness value of composite resin before and after its immersion in ethanol solution for 24 h, have been used to assess crosslink density [1, 18] instead of performing complex differential scanning calorimetry tests [19]. The vulnerability of the polymer to softening can be clarified by the solvent capability of diffusion in the composite resin and bonding to the polymer chains replacing the interchain secondary bonds. Thereby, the solvent jeopardizes the polymer chains entanglement and promotes dissolution of the residual monomer units. Such solvent, in the same time, cannot beat primary covalent crosslinks; thus the molecules of the polymer cannot be carried off into solution. The capability of a specific solvent to soften a material relies on their relative polarities [20, 21]. Substances that have comparable polarities will be equally solvable, but dissimilar polarities will make solubility progressively challenging. So, what is crucial is not the absolute value of the solubility limit, but its proximity to or difference from that of another substance The solubility parameters of the applied organic solvents E and MEK (26.2 *δ*/MPa^1/2^ and 19.3 *δ*/MPa^1/2^, resp.) are close to that of poly methyl methacrylate resin (18.6 *δ*/MPa^1/2^) [9]. In this study, two bulk fill composite resins have been investigated: the sculpt-able SureFill since 1990s with an overall survival rate of 94.76% in 1–5 years [22] and the relatively recent sculpt-able SonicFill 2010. Both are dimethacrylate composite resins that show closely matching solubility parameter to that of poly methyl methacrylate resin.

The hardness assessment of the two bulk fill composite resins was conducted to estimate the crosslink density resultant from storage in E and MEK. The results revealed that both solvents produced reduction in hardness of the investigated composite resins. Such reduction can be explained by the solvent-polymer interaction detailed earlier in this context. These results were also consistent with other studies that investigated the effects of organic solvents on composite resins [5, 15, 23]. However, the degradation was higher in MEK for both composite resins with significant value in SureFill. The same trend has been encountered during testing of viscoelastic stability, expressed by creep parameters, of different composite resins by Marghalani and Watts 2013 [5], the flexural properties of bulk fill composites by Marghalani 2015 [2], and finally the hardness and diametral tensile strength of bulk fill composites by Sunbul et al. 2016 [24]. Hence, this behavior could be considered an indication of the higher resemblance of the solubility parameters of MEK and the dimethacrylate monomer systems of the tested composite resins than in case of E. Therefore the assumption that MEK would be an efficient alternative for E in assessment of crosslink density is accepted.

SureFill showed significant higher crosslink density than SonicFill where the former showed less decrease in Vickers hardness number after being stored in FSOS. It can be speculated that the polymeric network of SureFill consists of more crosslinked chains rather than the linear polymeric structure present in SonicFill. Such linear polymeric structure can encourage the diffusion of solvent inside as stated by Ferracane 2006 [12]. The degree of disintegration is apparently related to the interaction of the solvents with the constituent oligomers, which are the same in both investigated bulk fill composites except for UDMA in SureFill and BIS-EMA in SonicFill (Table 1).

The higher resistance of SureFill to softening could be attributed to the presence of UDMA. The latter is an aliphatic flexible oligomer owing to the presence of flexible urethane linkage instead of the stiff iso-propylidene-di-phenoxy center core present in both Bis-GMA and its analog Bis-EMA. This flexibility allowed free initial mobility of UDMA ensuring the close proximity of the radical species, thus creating multiple centers of polymer growth (crosslink centers). In addition, the opportunity of chain transfer reactions through the imino group should be responsible for more rapid rate of polymerization in SureFill, evident in network formation prior to diffusion-controlled propagation stage [25–27]. Hence, the faster polymerization reaction rate is usually accompanied by formation of a more branched and crosslinked polymer network [1, 11].

Goņalves et al. 2009 [28] and Cornelio et al. 2014 [29] attributed the higher degree of conversion, achieved in their tentative composite resin formulations when the content of Bis-EMA was increased, to the lower initial viscosity. But this lower viscosity seemed to slow down the rate of polymerization thus delaying the onset of autoacceleration, which is responsible for creating numerous foci of polymer growth (crosslink centers). Thus, a linear and less branched polymer network is obviously expected to be the final product in these experimental composite formulations.

Moreover, one should emphasize that the higher crosslink density of SureFill has overshadowed the relatively higher hydrophilic nature of its UDMA oligomer due to the presence of urethane group (-NHCOO-) when compared to BIS-EMA with its ether group (-O-) of lower hydrophilicity. This could be supported by the study of Pfeifer et al. 2009, who concluded that experimental composite resins composed of Bis-GMA : TEGDMA : UDMA offered the best compromise between degree of conversion from one side and flexural properties, fracture toughness, and susceptibility to ethanol degradation from the other side.

In addition to the impact of the monomers, the particulate filler also provides an essential part in defining the resistance to the plasticizing effect of organic solvents. An increase in the filler loading is most probably associated with higher resistance of the tested composite resins to degradation. Surprisingly, SonicFill displayed higher deterioration after conditioning in the organic solvents in spite of its relatively higher filler loading, Table 1. On the other hand, SureFill features a specially designed interlocking particle technology that could present obstacle against the attack of food simulating organic softening agents [30]. Therefore, it seems that the pattern of the filler distribution, mainly short interfiller spacing, was responsible for slowing down the percolation effect of plasticizing agents on SureFill.

## 5. Conclusion

Within the limitation of this investigation, the following can be concluded:

1. The crosslink density of dimethacrylate-based bulk fill composite resins can be evaluated using either ethanol or methyl ethyl ketone food simulating organic solvents with expected higher values for the latter.
2. SureFill has higher crosslink density than SonicFill composite resin in both ethanol and methyl ethyl ketone food simulating organic solvents.

## Figures and Tables

**Table 1:** Technical specifications of the tested materials according to their manufacturers.

Composite resin	SonicFill (Sculpt-able)	SureFill (Sculpt-able)
Manufacturer	Kerr Corporation, CA, USA/Kavo Germany	Dentsply/Caulk (USA)

Increment thickness (mm)	5 mm	5 mm

Monomer	Bis-GMA Bis-EMA TEGDMA	Bis-GMA, UDMA, TEGDMA

Photoinitiator/coinitiators	Camphorquinone/tertiary amine	Camphorquinone/tertiary amine

Filler type	SiO_2_, Barium aluminosilicate glass oxides	Barium fluoro alumino borosilicate glass blend of fumed silica

Filler loading (wt%)	83.5	82

Filler loading (vol%)	Not declared	66

**Table 2:** Mean hardness number and crosslink density of the tested bulk fill composite resins after dry storage, storage in 75% ethanol, and storage in 100% methyl ethyl ketone.

Bulk fill composite resin	Dry storage	Storage in 75% E	Storage in 100% MEK	*P* value^¶^
SureFill				
Vickers hardness number (SD)	82.4 (2.4)	76.6 (2.1)	74.6 (1.7)	
Crosslink density (SD)	—	7.0 (1.4)	9.4 (2.5)	0.02^*∗*^

SonicFill				
Vickers hardness number (SD)	99.0 (1.3)	88.5 (1.6)	87.2 (1.8)	
Crosslink density (SD)	—	10.5 (1.8)	11.9 (1.3)	0.06

*P* value^€^		<0.0001^*∗*^	0.02^*∗*^	

SD: standard deviation.

^¶^ *P* value of *t*-test comparing differences after using solvents in each material.

^€^ *P* value of *t*-test comparing the two materials when each solvent was used.

^*∗*^Statistically significant difference at *P* ≤ 0.05.
